# New Sorafenib Derivatives: Synthesis, Antiproliferative Activity Against Tumour Cell Lines and Antimetabolic Evaluation

**DOI:** 10.3390/molecules17011124

**Published:** 2012-01-23

**Authors:** Željka Babić, Maja Crkvenčić, Zrinka Rajić, Ana-Matea Mikecin, Marijeta Kralj, Jan Balzarini, Mariya Petrova, Jos Vanderleyden, Branka Zorc

**Affiliations:** 1 Faculty of Pharmacy and Biochemistry, University of Zagreb, A. Kovačića 1, HR-10000 Zagreb, Croatia; Email: zeljka.babic@sk.t-com.hr (Ž.B.); maja.crkvencic@kr.t-com.hr (M.C.); zrajic@pharma.hr (Z.R.); 2 Division of Molecular Medicine, Rudjer Bošković Institute, Bijenička cesta 54, HR-10000 Zagreb, Croatia; Email: amikecin@irb.hr (A.M.M.); marijeta.kralj@irb.hr (M.K.); 3 Rega Institute for Medical Research, Katholieke Universiteit Leuven, Minderbroedersstraat 10, B-3000 Leuven, Belgium; Email: Jan.Balzarini@rega.kuleuven.be; 4 Centre of Microbial and Plant Genetics, Katholieke Universiteit Leuven, Kasteelpark Arenberg 20, Bus 2300, B-3001 Heverlee, Belgium; Email: Mariya.Petrova@biw.kuleuven.be (M.P.); Jozef.Vanderleyden@biw.kuleuven.be (J.V.)

**Keywords:** sorafenib, amides, cytostatic activity, antimetabolic activity, Caco-2 cells permeability

## Abstract

Sorafenib is a relatively new cytostatic drug approved for the treatment of renal cell and hepatocellular carcinoma. In this report we describe the synthesis of sorafenib derivatives **4a**–**e** which differ from sorafenib in their amide part. A 4-step synthetic pathway includes preparation of 4-chloropyridine-2-carbonyl chloride hydrochloride (**1**), 4-chloro-pyridine-2-carboxamides **2a**–**e**, 4-(4-aminophenoxy)-pyridine-2-carboxamides **3a**–**e** and the target compounds 4-[4-[[4-chloro-3-(trifluoromethyl)phenyl]carbamoylamino]-phenoxy]-pyridine-2-carboxamides **4a**–**e**. All compounds were fully chemically characterized and evaluated for their cytostatic activity against a panel of carcinoma, lymphoma and leukemia tumour cell lines. In addition, their antimetabolic potential was investigated as well. The most prominent antiproliferative activity was obtained for compounds **4a**–**e** (IC_50_ = 1-4.3 μmol·L^−1^). Their potency was comparable to the potency of sorafenib, or even better. The compounds inhibited DNA, RNA and protein synthesis to a similar extent and did not discriminate between tumour cell lines and primary fibroblasts in terms of their anti-proliferative activity.

## 1. Introduction

Sorafenib, 4-[4-[[4-chloro-3-(trifluoromethyl)phenyl]carbamoylamino]phenoxy]-*N*-methylpyridine-2-carboxamide, is an oral multikinase inhibitor that inhibits cell surface tyrosine kinase receptors (e.g., vascular endothelial growth factor receptors and platelet-derived growth factor receptor-beta) and downstream intracellular serine/threonine kinases (e.g., Raf-1, wild-type B-Raf and mutant B-Raf); these kinases are involved in tumour cell proliferation and tumour angiogenesis [1,2,3]. Sorafenib is approved for the treatment of advanced renal cell carcinoma and hepatocellular carcinoma [4,5]. Clinical trials to use sorafenib for non-responsive thyroid cancer and glioblastoma are in progress as well. However, both median survival and time to progression in sorafenib therapy showed only a 3-month improvement in patients who received sorafenib compared to placebo [6,7]. These facts point to sorafenib as an interesting lead compound for further derivatization in order to find a more effective drug. The present study is focused on the synthesis of new sorafenib derivatives bearing the same diarylurea moiety as sorafenib and differing from the parent drug in the amide part. The newly prepared compounds are more lipophilic in an attempt to increase uptake and/or accumulation of the drug in tissues [8]. Here we report their synthesis and in vitro evaluation of their cytostatic activity against several carcinoma cell lines.

## 2. Results and Discussion

### 2.1. Chemistry

**Scheme 1:**
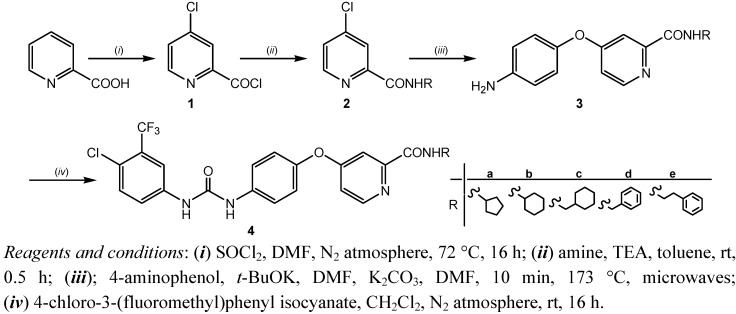
Synthesis of sorafenib derivatives **4a–e**.

A four step synthesis of sorafenib amide analogues **4a**–**e** is described. Scheme 1 outlines the general preparative route. The starting material, picolinic acid, was first converted to the acid chloride using thionyl chloride [9]. Under the reaction conditions employed (72 °C, 16 h, nitrogen atmosphere), chlorination of the pyridine ring at the *para-*position occurred as well and the final product was 4-chloropyridine-2-carbonyl chloride hydrochloride (**1**). Amidation of the acid chloride **1** with five different amines (cyclopentylamine, cyclohexylamine, cyclohexylmethylamine, benzylamine and phenylethylamine) gave amides **2a**–**e**. The amidation step was performed at room temperature in the presence of triethylamine as HCl acceptor. The selected amines were chosen on the basis of their lipophilicity and limited number of possible conformations, providing more lipohilic and rigid final compounds **4**. The reactions of acid chloride **1** with the title amines gave the amides in good yields. Amide **2c** was also prepared from picolinic acid methyl ester (methyl 4-chloropyridine-2-carboxylate) and cyclohexylmethylamine. However, analogous reactions with cyclopentylamine and cyclohexyl-amine afforded the corresponding amides in poor yields.

In the next reaction step products **2a**–**e** were coupled with 4-aminophenol to give the ethers **3a**–**e**. The ether side chain was introduced using potassium *tert*-butoxide in the presence of potassium carbonate (2 h, 80 °C). Under basic conditions employed, the alkoxide derived from 4-aminophenol was a stronger nucleophile than the amino group, therefore the main product was the ether, not the secondary amine. The reaction time was shortened to 10 min when the ether formation reactions were run in a microwave reactor (*P* = 150 W, *t* = 173 °C). Ethers **3a**–**e** readily reacted with isocyanate to give the final products **4a**–**e**. Urea bond formation was performed at room temperature, in nitrogen atmosphere, using a slight excess of 4-chloro-3-(fluoromethyl)phenyl isocyanate.

All intermediates and final amides **4a**–**e** were isolated and fully characterized by elemental analysis and usual spectroscopic methods (IR, ^1^H-, ^13^C-NMR and MS). The spectral data are consistent with the proposed structures (Table 1).

**Table 1:** ^1^H and ^13^C-NMR spectra of compounds **2a–e**, **3a–e**, and **4a–e**. 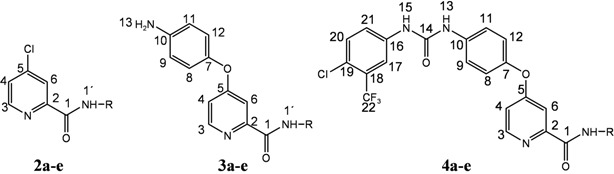

Compd.	R	^1^H and ^13^C-NMR spectra/*δ* ppm, *J*/Hz
**2a**	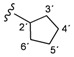	8.62 (d, 1H, 3, *J* = 5.4), 8.58 (d, 1H, 1', *J =* 7.6), 8.02 (d, 1H, 6, *J =* 1.8), 7.75 (dd, 1H, 4, *J =* 2.1, *J =* 3.2), 4.31–4.19 (m, 1H, 2'), 1.94–1.53 (m, 8H, 3'–6')
		161.26 (1), 151.02 (2), 149.02 (3), 143.61 (5), 125.36 (6), 120.98 (4), 49.82 (2'), 31.12 (3', 6'), 22.62 (4', 5')
**2b**	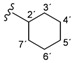	8.61 (d, 1H, 3, *J =* 5.2), 8.48 (d, 1H, 1', *J =* 8.1), 8.01 (d, 1H, 6, *J =* 1.9), 7.74 (dd, 1H, 4, *J =* 1.9, *J =* 3.2), 3.80–3.74 (m, 1H, 2'), 1.79–1.16 (m, 10H, 3'–7')
		161.54 (1), 151.90 (2), 149.91 (3), 144.53 (5), 126.28 (6), 121.90 (4), 48.13 (2'), 32.03 (3', 7'), 25.03 (5'), 24.73 (4', 6')
**2c**	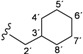	8.82 (t, 1H, 1', *J =* 8.8), 8.62 (d, 1H, 3, *J =* 8.6), 8.02 (d, 1H, 6, *J =* 1.8), 7.76 (dd, 1H, 4, *J =* 2.1, *J =* 3.1), 3.16 (t, 2H, 2', *J =* 6.6), 1.68–0.97 (m, 11H, 3'–8')
		163.08 (1), 152.37 (2), 150.46 (3), 145.00 (5), 126.76 (6), 122.39 (4), 45.52 (2'), 30.86 (4', 8'), 26.50 (6'), 25.81 (5', 7')
**2d**	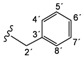	9.40 (t, 1H, 1', *J =* 6.2), 8.62 (d, 1H, 3, *J =* 5.5), 8.01 (d, 1H, 6, *J =* 2.0), 7.77 (dd, 1H, 4, *J =* 2.1, *J =* 3.3), 7.33–7.21 (m, 5H, arom. 4'–8'), 4.50 (d, 2H, 2', *J =* 6.1)
		163.26 (1), 152.23 (2), 150.57 (3), 145.04 (5), 139.76 (3'), 128.72 (4', 8'), 127.83 (5', 7'), 126.94 (6'), 125.36 (6), 120.98 (4), 42.98 (2')
**2e**	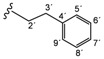	8.88 (t, 1H, 1', *J =* 5.6), 8.61 (d, 1H, 3, *J =* 5.0), 8.01 (d, 1H, 6, *J =* 1.8), 7.73 (dd, 1H, 4, *J =* 2.1, *J =* 3.2), 3.55–3.52 (q, 2H, 2', *J* = 7.0), 2.86 (t, 2H, 3', *J =* 7.5)
		162.50 (1), 151.73 (2), 149.98 (3), 144.52 (5), 139.24 (4'), 128.54 (5', 9'), 128.31 (6', 8'), 126.33 (6), 126.08 (7'), 121.86 (4), 40.44 (2'), 34.97 (3')
**3a**	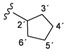	8.46 (d, 1H, 3, *J =* 2.5), 8.44 (d, 1H, 1', *J =* 8.5), 7.34 (d, 1H, 6, *J =* 2.4), 7.08 (dd, 1H, 4, *J =* 2.6, *J =* 2.9), 6.85 (d, 2H, 8, 12, *J =* 8.9), 6.66 (d, 2H, 9, 11, *J =* 8.9), 5.16 (s, 2H, 13), 4.22–4.15 (m, 1H, 2'), 1.99–1.53 (m, 8H, 3'–6')
		167.30 (5), 163.31 (1), 152.74 (2), 150.51 (3), 147.32 (7), 143.37 (10), 121.99 (8, 12), 115.36 (9, 11), 114.23 (6), 108.86 (4), 51.04 (2'), 32.56 (3', 6'), 23.95 (4', 5')
**3b**	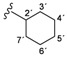	8.45 (d, 1H, 3, *J =* 5.7), 8.37 (d, 1H, 1', *J =* 8.5), 7.34 (d, 1H, 6, *J =* 2.4), 7.08 (dd, 1H, 4, *J =* 2.6, *J =* 2.9), 6.85 (d, 2H, 8, 12, *J =* 8.9), 6.65 (d, 2H, 9, 11, *J =* 8.9), 5.17 (s, 2H, 13), 3.77–3.68 (m, 1H, 2'), 1.78–1.04 (m, 10H, 3'–7')
		167.31 (5), 162.70 (1), 152.77 (2), 150.52 (3), 147.30 (7), 143.38 (10), 121.98 (8, 12), 115.37 (9, 11), 114.26 (6), 108.92 (4), 48.38 (2'), 32.58 (3', 7'), 25.54 (5'), 25.18 (4', 6')
**3c**	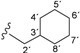	8.65 (t, 1H, 1', *J =* 6.2), 8.45 (d, 1H, 3, *J =* 5.6), 7.34 (d, 1H, 6, *J =* 2.3), 7.07 (dd, 1H, 4, *J =* 2.6, *J =* 3.1), 6.86 (d, 2H, 8, 12, *J =* 8.6), 6.65 (d, 2H, 9, 11, *J =* 8.9), 5.15 (s, 2H, 13), 3.12 (t, 2H, 2', *J =* 6.6), 1.66–0.84 (m, 11H, 3'–8')
		167.31 (5), 163.76 (1), 152.78 (2), 150.54 (3), 147.32 (7), 143.37 (10), 122.00 (8, 12), 115.36 (9, 11), 114.19 (6), 108.90 (4), 45.39 (2'), 37.91 (3'), 30.86 (4', 8'), 26.50 (6'), 25.81 (5', 7')
**3d**	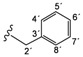	9.27 (t, 1H, 1', *J =* 6.4), 8.47 (d, 1H, 3, *J =* 5.6), 7.38 (d, 1H, 6, *J =* 2.4), 7.31–7.21 (m, 5H, 4'–8'), 7.08 (dd, 1H, 4, *J =* 2.7, *J* = 2.9), 6.85 (d, 2H, 8, 12, *J* = 8.8), 6.65 (d, 2H, 9, 11, *J* = 8.8), 5.16 (s, 2H, 13), 4.46 (d, 2H, 2', *J* = 6.4)
		167.32 (5), 163.96 (1), 152.63 (2), 150.66 (3), 147.33 (7), 143.36 (10), 139.89 (3'), 128.70 (4', 8'), 127.81 (5', 7'), 127.22 (6'), 122.01 (8, 12), 115.38 (9, 11), 114.35 (6), 109.09 (4), 42.91 (2')
**3e**	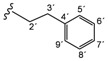	8.78 (t, 1H, 1', *J* = 6.0), 8.44 (d, 1H, 3, *J* = 5.5), 7.35 (d, 1H, 6, *J* = 2.6), 7.30–7.18 (m, 5H, 5'–9'), 7.08 (dd, 1H, 4, *J* = 2.6, *J* = 3.1), 6.86 (d, 2H, 8, 12, *J* = 8.7), 6.65 (d, 2H, 9, 11, *J* = 8.7), 5.17 (s, 2H, 13), 3.52 (q, 2H, 2', *J* = 7.0), 2.84 (t, 2H, 3', *J* = 7.5)
		166.80 (5), 163.23 (1), 152.14 (2), 150.09 (3), 146.84 (7), 142.83 (10), 139.29 (4'), 128.53 (5', 9'), 128.31 (6', 8'), 126.06 (7'), 121.51 (8, 12), 114.86 (9, 11), 113.73 (6), 108.42 (4), 39.55 (2'), 24.06 (3')
**4a**	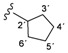	9.20 (s, 1H, 15), 8.98 (s, 1H, 13), 8.50 (d, 1H, 3, *J* = 5.4), 8.47 (d, 1H, 1', *J* = 6.7), 8.17 (s, 1H, 17), 7.69–7.58 (m, 4H, 20, 21, 8, 12), 7.38 (d, 1H, 6, *J* = 2.6), 7.18–7.14 (m, 3H, 4, 9, 11), 4.23–4.16 (m, 1H, 2'), 1.99–1.53 (m, 8H, 3'–6')
		166.47 (5), 163.21 (1), 152.94 (14), 152.92 (2), 150.75 (3), 148.36 (7), 139.80 (10), 137.52 (16), 132.44 (22), 124.00 (19), 123.58 (20), 122.54 (18), 121.86 (8, 12), 120.99 (9, 11), 117.36 (17), 117.28 (21), 114.59 (6), 109.20 (4), 51.07 (2'), 32.55 (3', 6'), 23.96 (4', 5')
**4b**	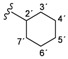	9.45 (s, 1H, 15), 9.19 (s, 1H, 13), 8.50 (d, 1H, 3, *J* = 5.6), 8.40 (d, 1H, 1', *J* = 8.6), 8.11 (s, 1H, 17), 7.68–7.58 (m, 4H, 20, 21, 8, 12), 7.39 (d, 1H, 6, *J* = 2.4), 7.18–7.14 (m, 3H, 4, 9, 11), 3.75–3.72 (m, 1H, 2'), 1.78–1.56 (m, 10H, 3'–7')
		166.49 (5), 162.61 (1), 152.99 (14), 152.93 (2), 150.77 (3), 148.31 (7), 139.85 (10), 137.56 (16), 132.47 (22), 123.93 (19), 123.45 (20), 122.46 (18), 121.87 (8, 12), 120.84 (9, 11), 117.21 (17), 117.13 (21), 114.59 (6), 109.31 (4), 48.42 (2'), 32.57 (3', 7'), 25.53 (5'), 25.18 (4',6')
**4c**	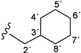	9.20 (s, 1H, 15), 8.97 (s, 1H, 13), 8.69 (t, 1H, 1', *J* = 6.1), 8.51 (d, 1H, 3, *J* = 5.5), 8.12 (s, 1H, 17), 7.69–7.58 (m, 4H, 20, 21, 8, 12), 7.38 (d, 1H, 6, *J* = 2.5), 7.18–7.14 (m, 3H, 4, 9, 11), 3.13 (t, 2H, 2', *J* = 6.4), 1.66–0.78 (m, 11H, 3'–8')
		166.49 (5), 163.66 (1), 152.94 (14), 152.92 (2), 150.78 (3), 148.35 (7), 139.80 (10), 137.52 (16), 132.45 (22), 124.04 (19), 123.58 (20), 122.58 (18), 121.90 (8, 12), 120.99 (9, 11), 117.36 (17), 117.29 (21), 114.57 (6), 109.22 (4), 45.41 (2'), 37.90 (3'), 30.86 (4', 8'), 26.50 (6'), 25.81 (5', 7')
**4d**	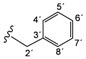	9.49 (s, 1H, 15), 9.31 (t, 1H, 1', *J* = 6.3), 9.22 (s, 1H, 13), 8.54 (d, 1H, 3, *J* = 5.6), 8.12 (s, 1H, 17), 7.65–7.58 (m, 4H, 20, 21, 8, 12), 7.42 (d, 1H, 6, *J* = 2.4), 7.31–7.16 (m, 8H, 4, 9, 11, 4'–8'), 4.46 (d, 2H, 2', *J* = 6.3)
		166.50 (5), 163.85 (1), 153.00 (14), 152.80 (2), 150.91 (3), 148.27 (7), 139.86 (10), 137.60 (16), 132.48 (22), 128.70 (4', 8'), 127.83 (5', 7'), 127.23 (6'), 126.66 (3'), 123.99 (19), 123.43 (20), 122.57 (18), 121.91 (8, 12), 120.83 (9, 11), 117.18 (17), 117.11 (21), 114.67 (6), 109.45 (4), 42.92 (2')
**4e**	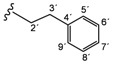	9.20 (s, 1H, 15), 8.98 (s, 1H, 13), 8.81 (t, 1H, 1', *J* = 5.8), 8.50 (d, 1H, 3, *J* = 5.6), 8.12 (s, 1H, 17), 7.68–7.58 (m, 4H, 20, 21, 8, 12), 7.39 (d, 1H, 6, *J* = 2.3), 7.31–7.14 (m, 8H, 4, 9, 11, 4'–8'), 3.51 (q, 2H, 2', *J* = 6.9), 2.84 (t, 2H, 3', *J* = 7.4)
		166.48 (5), 163.62 (1), 152.94 (14), 152.81 (2), 150.83 (3), 148.33 (7), 139.79 (10), 137.53 (16), 132.46 (22), 129.03 (5', 9'), 128.81 (6', 8'), 126.73 (4'), 126.57 (7'), 125.11 (19), 123.60 (20), 122.58 (18), 121.90 (8, 12), 121.01 (9, 11), 117.60 (17, 21), 114.60 (6), 109.24 (4), 40.83 (2'), 35.50 (3')

### 2.2. Biological Evaluation

#### 2.2.1. Cytostatic Activity

The experiments were carried out on six human tumour and one murine cell lines, which are derived from five solid tumour types and two (one murine and one human) suspension tumour cell lines. The following cell lines were used: HCT 116 (colon carcinoma), SW 620 (colon carcinoma), MCF-7 (breast carcinoma), H 460 (lung carcinoma), L1210 (murine leukemia), CEM (human lymphoma) and HeLa (cervix carcinoma). The results of the cytostatic effect of the tested compounds are presented in Table 2. Compounds **2a**–**e** and **3a**–**c** showed no, or rather modest antiproliferative effect, only at the maximal tested concentration (10^-4^ M). Compounds **3d** and **3e** with a benzene ring in the amide moiety, exerted a somewhat stronger (but still rather poor) cytostatic effect (IC_50_ ≈ 20-90 µM). In contrast, compounds **4a**–**e** showed the most prominent effect with IC_50_ values in the lower micromolar range (IC_50_ from 1 to 4.3 mM, with an average of 2.6 ± 1.6), but without substantial selectivity between the tumour cell lines. The compounds were also equally cytostatic against primary human embryonic lung (HEL) fibroblast cells (Table 2). The obtained data clearly point to the obligatory presence of the [4-chloro-3-(trifluoromethyl)phenyl]carbamoyl group being crucial for high potency. Also, the **4a**–**e** series of compounds are far more lipophilic than the **2** and **3** series of compounds, which may be related to their increased cytostatic potential. The cytostatic activities of compounds **4a**–**e** are comparable with the data obtained for sorafenib [10] or even better (median IC_50_ value for sorafenib is 4.3 mM; twenty of 23 cell lines had values between 1.0 and 10.0 mM), which indicate that the pyridine-2-carboxamide modifications had a minor effect on the antiproliferative activity. Log *P* of sorafenib is 3.76, while amides **4a**–**e** have log *P* values between 4.89 and 5.77 (Table 2) [11].

**Table 2:** IC_50_ values of compounds **2a–e**, **3a–e**, and **4a–e**^ a^.

Compd.	log P (Clog P) ^b^	Tumour cell growth/IC50 (µmol·L^−1^)							
		HCT 116	SW 620	MCF-7	H 460	L1210	CEM	HeLa	HEL
**2a**	1.95 (2.72)	>100	>100	>100	≥100	>100	>100	>100	>100
**2b**	2.37 (3.28)	>100	>100	≥100	35 ± 12	>100	>100	>100	>100
**2c**	2.80 (3.90)	≥100	≥100	≥100	>100	>100	>100	>100	>100
**2d**	2.55 (3.22)	≥100	71 ± 28	≥100	>100	≥100	≥100	>100	>100
**2e**	2.83 (3.35)	≥100	>100	57 ± 46	>100	>100	>100	>100	>100
**3a**	2.13 (2.80)	54 ± 21	≥100	53 ± 40	>100	121 ± 50.5	≥100	77 ± 3.4	>100
**3b**	2.54 (3.36)	37 ± 1	≥100	≥100	65 ± 33	234 ± 4.5	>100	>100	>100
**3c**	2.97 (3.98)	≥100	≥100	≥100	>100	163 ± 147	>100	>100	>100
**3d**	2.73 (3.30)	29 ± 4	37 ± 6	29 ± 13	42 ± 9	66 ± 3.1	94 ± 2.2	94 ± 1.9	>100
**3e**	3.01 (3.43)	25 ± 2	20 ± 7	34 ± 17	20 ± 0.0	63 ± 8.9	42 ± 3.0	39 ± 3.0	>100
**4a**	4.89 (6.93)	3.0 ± 0.5	2.0 ± 0.4	4.0 ± 1.0	1.0 ± 0.0	4.2 ± 0.8	4.2 ± 0.6	3.1 ± 0.6	2.8 ± 0.0
**4b**	5.31 (7.49)	3.0 ± 0.8	3.0 ± 0.2	2.0 ± 0.6	2.0 ± 0.0	3.2 ± 1.7	3.6 ± 0.9	3.0 ± 0.2	3.2 ± 0.2
**4c**	5.73 (8.11)	3.0 ± 1.0	3.0 ± 0.4	2.0 ± 0.1	2.0 ± 0.2	1.8 ± 0.3	2.0 ± 0.5	2.7 ± 1.8	2.9 ± 0.0
**4d**	5.49 (7.42)	2.0 ± 0.8	2.0 ± 0.08	2.0 ± 0.2	2.0 ± 0.2	2.0 ± 0.4	4.3 ± 0.4	2.6 ± 0.6	3.1 ± 0.2
**4e**	5.77 (7.55)	3.0 ± 0.7	2.0 ± 0.4	2.0 ± 0.1	2.0 ± 0.2	2.2 ± 0.2	2.5 ± 0.9	1.7 ± 0.2	4.6 ± 0.36
Sorafenib	3.76 (5.46)	3.0 ± 0.8	6.0 ± 1.0	3.0 ± 0.7	3.0 ± 0.8	4.1 ± 0.0	3.4 ± 0.5	3.7 ± 0.6	7.5 ± 0.7

^a^ IC_50_, the concentration that causes 50% growth inhibition; ^b^ Lipophilicity was theoretically calculated by the CLOGP Programme of Biobyte Corp. [11].

#### 2.2.2. Antimetabolic Activity

A variety of compounds (**2a**, **3a**, **4a** with a cycloalkyl substituent directly attached to nitrogen atom, **2c**, **3c**, **4c** with a cycloalkyl bound to nitrogen through a short spacer and **2e**, **3e**, **4e** with an aromatic substituent) have been investigated on their inhibitory activity against DNA, RNA and protein synthesis in CEM cell cultures [12]. The compounds were equally inhibitory to dThd (DNA synthesis), Urd (RNA synthesis) and Leu (protein synthesis) incorporation into TCA-insoluble cell material. The **2** series of compounds were least inhibitory whereas the **4** series were most inhibitory (IC_50_ in the lower micromolar range). Their antimetabolic IC_50_ values closely corresponded to their antiproliferative IC_50_ values. They also behaved as sorafenib in terms of antimetabolic potential (Table 3).

**Table 3:** Antimetabolic activity of tested compounds in CEM cell cultures.

CEM	IC_50_ (µmol·L^−1^)		
	[CH_3_-^3^H]dThd	[5-^3^H]Urd	[4,5-^3^H]Leu
**2a**	>100	>100	>100
**2c**	>100	>100	>100
**2e**	>100	>100	>100
**3a**	>100	>100	>100
**3c**	>100	92.1 ± 43.0	70.6 ± 27.6
**3e**	>100	49.9 ± 17.9	>100
**4a**	12.5 ± 6.7	7.7 ± 2.1	6.1 ± 0.7
**4c**	20.1 ± 10.9	12.2 ± 8.4	27.4 ± 12.7
**4e**	12.7 ± 9.0	6.6 ± 1.8	11.8 ± 7.7
Sorafenib	8.6 ± 1.1	7.1 ± 0.2	12.0 ± 7.0

^a^ 50% Inhibitory concentration or compound concentration required to inhibit tritated dThd, Urd or Leu into TCA-insoluble cell materials.

#### 2.2.3. Uptake and Efflux of Tested Compounds in CaCo-2 Cell Cultures

**Figure 1:**
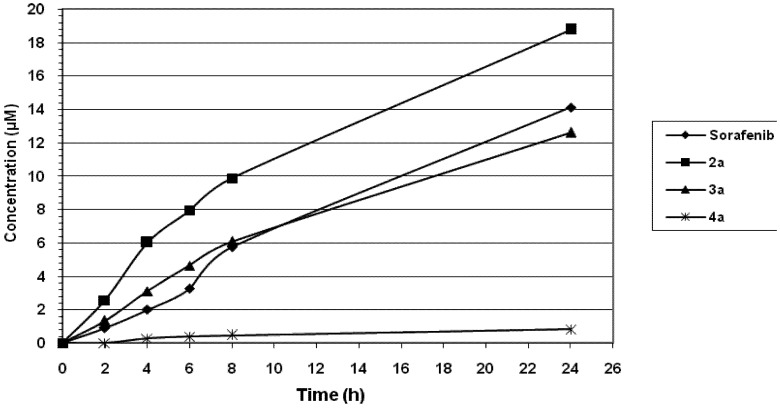
Release of the tested compounds at the basolateral side by CaCo-2 cell cultures. Hundred micromolar concentrations of the tested compounds were added at the apical side of confluent CaCo-2 cell cultures, and the appearance of the intact tested compound at the basolateral side was measured by HPLC analysis.

Derivatives **2a**, **3a** and **4a** have been examined for their efficiency of uptake at the apical site and subsequent delivery at the basolateral site of colon carcinoma CaCo-2 cell cultures [13]. This model system can be used to predict the potential of a drug to show oral bioavailability. Sorafenib was included as the reference compound. Whereas **2a** and **3a** showed a higher drug delivery at the basolateral site than sorafenib in function of exposure time, **4a** performed less efficient than the reference compound (Figure 1). The molecular basis of this phenomenon is yet unclear.

## 3. Experimental Section

### 3.1. Chemistry

#### 3.1.1. General

Melting points were determined on a Stuart Melting Point Apparatus SMP3 and were uncorrected. IR spectra were recorded on a FTIR Perkin Elmer Paragon 500 spectrometer. ^1^H- and ^13^C-NMR spectra were recorded on a Bruker AV-600 spectrometer operating at 300.13 and 75.47 MHz for the ^1^H and ^13^C nuclei, respectively. Samples were measured in DMSO-*d*_6_ solutions at 20 °C in 5 mm NMR tubes. Chemical shifts (*δ*) were referred to TMS. Mass spectra were taken on a HPLC-MS/MS (HPLC, Agilent Technologies 1200 Series; MS, Agilent Technologies 6410 Triple Quad). CEM Discover microwave reactor was used for microwave reactions. Precoated Merck silica gel 60 F_254_ plates (Merck, Germany) and solvent systems petrolether/ethyl acetate/methanol (3:1:0.5; 3:1:0.25 or 3:1:0.15), cyclohexane/ethyl acetate (1:1), petrolether/ethyl acetate (1:1), ethyl acetate and dichloromethane/methanol (9:1 or 9.5:0.5), were used for thin-layer chromatography. Spots were visualized by short-wave UV light and iodine vapour. Column chromatography was performed on silica gel (0.063–0.200 mm), with petroleum ether/ethyl acetate/methanol (3:1:0.5, 3:1:0.25 or 3:1:0.15) or dichloromethane/methanol (9:1). Preparative chromatography was performed on silica gel preparative plates using dichloromethane/methanol (9:1 or 9.5:0.5) as eluents. Picolinic acid, 4-amino-phenol and 4-chloro-3-(fluoromethyl)phenyl isocyanate were purchased from Acros Organics, cyclopentylamine, cyclohexylamine, cyclohexylmethylamine, benzylamine, phenylethylamine and potassium *tert*-butoxyde from Aldrich, and potassium carbonate from Kemika. Anhydrous solvents (DMF, CH_2_Cl_2_, toluene) were prepared prior to use. Human cell lines for cytostatic activity were obtained from American Type Culture Collection.

*4-Chloropyridine-2-carbonyl chloride hydrochloride* (**1**). Compound **1** was prepared from picolinic acid (1.231 g, 10 mmol) and thionyl chloride (5 mL) following procedure previously described [9]. The crude product **1** was used in the next step without further purification.

#### 3.1.2. General Method for the Synthesis of 4-chloropyridine-2-carboxamides **2a–e**

To a cold solution of chloride **1** (1.760 g, 10 mmol) in anhydrous toluene (10 mL) a solution of appropriate amine (10 mmol) and triethylamine (2.02 g, 20 mmol) in toluene (5 mL) was added dropwise. The reaction mixture was stirred at room temperature for 30 min and then extracted 3 times with saturated NaCl solution. The organic phase was dried over anhydrous Na_2_SO_4_, filtered and evaporated under reduced pressure. The crude products **2a**–**e** were purified by column chromatography.

*4-Chloro-N-cyclopentylpyridine-2-carboxamide* (**2a**). From the reaction of chloride **1** (1.760 g, 10 mmol) and cyclopentylamine (0.086 g, 10 mmol) at room temperature for 0.5 h and column chromatography (mobile phase petroleum ether/ethyl acetate/methanol 3:1:0.5 and petroleum ether/ethyl acetate 1:1) 1.621 g (72%) of **2a** was obtained; mp 79-80 °C; IR (KBr): *υ*_max_ 3328, 3064, 2961, 2870, 1666, 1575, 1556, 1519, 732 cm^–1^. MS/MS *m/z* 225.1 (M+H)^+^. Anal. (C_11_H_13_ClN_2_O) C, H, N.

*4-Chloro-N-cyclohexylpyridine-2-carboxamide* (**2b**). From the reaction of chloride **1** (1.760 g, 10 mmol) and cyclohexylamine (0.099 g, 10 mmol) at room temperature for 0.5 h and column chromatography (mobile phase petroleum ether/ethyl acetate/methanol 3:1:0.15) 1.243 g (52%) of **2b** was obtained; mp 116-119 °C; IR (KBr): *υ*_max_ 3330, 2930, 2853, 1650, 1578, 1554, 1534, 736 cm^–1^. MS/MS *m/z* 239.1 (M+H)^+^. Anal. (C_12_H_15_ClN_2_O) C, H, N.

*4-Chloro-N-cyclohexylmethylpyridine-2-carboxamide* (**2c**). From the reaction of chloride **1** (1.760 g, 10 mmol) and cyclohexylmethylamine (1.132 g, 10 mmol) at room temperature for 0.5 h and column chromatography (mobile phase petroleum ether/ethyl acetate/methanol 3:1:0.15) 0.999 g (40%) of **2c** was obtained; mp 107-108 °C; IR (KBr): *υ*_max_ 3341, 3089, 2926, 2855, 1649, 1537, 736 cm^–1^. MS/MS *m/z* 253.1 (M+H)^+^. Anal. (C_13_H_17_ClN_2_O) C, H, N.

*4-Chloro-N-benzylpyridine-2-carboxamide* (**2d**). From the reaction of chloride **1** (1.760 g, 10 mmol) and benzylamine (1.072 g, 10 mmol) at room temperature for 0.5 h and column chromatography (mobile phase petroleum ether/ethyl acetate/methanol 3:1:0.25) 1.577 g (64%) of **2d** was obtained; mp 70-72 °C; IR (KBr): *υ*_max_ 3326, 1649, 1578, 1552, 1536, 736, 725 cm^–1^. MS/MS *m/z* 269.1 (M+Na)^+^. Anal. (C_13_H_11_ClN_2_O) C, H, N.

*4-Chloro-N-phenylethylpyridine-2-carboxamide* (**2e**). From the reaction of chloride **1** (1.760 g, 10 mmol) and phenylethylamine (1.212 g, 10 mmol) at room temperature for 0.5 h and column chromatography (mobile phase petroleum ether/ethyl acetate/methanol 3:1:0.15) 1.404 g (54%) of **2e** was obtained; mp 60-62 °C; IR (KBr): *υ*_max_ 3346, 1657, 1556, 1531, 733, 700 cm^–1^. MS/MS *m/z* 261.1 (M+H)^+^. Anal. (C_14_H_13_ClN_2_O) C, H, N.

#### 3.1.3. General Method for the Synthesis of 4-(4-aminophenoxy)-pyridine-2-carboxamides **3a–e**

A suspension of *t*-BuOK (0.056 g, 0.5 mmol) and 4-aminophenol (0.054 g, 0.5 mmol) in anhydrous DMF (5 mL) was stirred at room temperature for 30 min. The corresponding 4-chloropicolinamide **2a**–**e** (0.5 mmol) and potassium carbonate (0.034 g, 0.25 mmol) were added. The reaction mixture was stirred in microwave reactor for 10 min (*P* = 150 W, *t* = 173 °C). DMF was evaporated under reduced pressure. The residue was dissolved in ethyl acetate end extracted with saturated NaCl solution. The organic phase was dried over anhydrous Na_2_SO_4_, filtered and evaporated under reduced pressure. The crude products **3a**–**e** were purified by column chromatography or by recrystallization.

*4-(4-Aminophenoxy)-N-cyclopentylpyridine-2-carboxamide* (**3a**). From the reaction of amide **2a** (0.112 g, 0.5 mmol) and purification by column chromatography [mobile phase dichloromethane/methanol (9:1)] 0.069 g (46%) of **3a** was obtained; viscous oil; IR (KBr): *υ*_max_ 3353, 2958, 2870, 1666, 1608, 1590, 1521, 1502, 1470, 1284, 1198, 836 cm^–1^. MS/MS *m/z* 298.2 (M+H)^+^. Anal. (C_17_H_19_N_3_O_2_) C, H, N.

*4-(4-Aminophenoxy)-N-cyclohexylpyridine-2-carboxamide* (**3b**). From the reaction of amide **2b** (0.119 g, 0.5 mmol) and recrystallization from methanol/water 0.075 g (48%) of **3b** was obtained; mp 150-153 °C. IR (KBr): *υ*_max_ 3357, 3316, 2928, 2855, 1660, 1566, 1523, 1506, 839 cm^–1^. MS/MS *m/z* 312.2 (M+H)^+^. Anal. (C_18_H_21_N_3_O_2_) C, H, N.

*4-(4-Aminophenoxy)-N-cyclohexylmethylpyridine-2-carboxamide* (**3c**). From the reaction of amide **2c** (0.126 g, 0.5 mmol) and purification by column chromatography (mobile phase petroleum ether/ethyl acetate/methanol 3:1:0.15) 0.075 g (46%) of **3c** was obtained; mp 135-137 °C; IR (KBr): *υ*_max_ 3390, 3364, 3194, 2924, 2838, 1680, 1526, 1504, 1299, 1195, 844 cm^–1^. MS/MS *m/z* 326.2 (M+H)^+^. Anal. (C_19_H_23_N_3_O_2_) C, H, N.

*4-(4-Aminophenoxy)-N-benzylpyridine-2-carboxamide* (**3d**). From the reaction of amide **2d** (0.123 g, 0.5 mmol) and purification by column chromatography (mobile phase petroleum ether/ethyl acetate/methanol 3:1:0.5) 0.121 g (76%) of **3d** was obtained; viscous oil; IR (KBr): *υ*_max_ 3348, 3062, 2925, 1665, 1590, 1566, 1526, 1501, 1468, 1261, 1197, 834, 699 cm^–1^. Anal. (C_19_H_17_N_3_O_2_) C, H, N.

*4-(4-Aminophenoxy)-N-phenylethylpyridine-2-carboxamide* (**3e**). From the reaction of amide **2e** (0.130 g, 0.5 mmol) and purification by column chromatography (mobile phase petroleum ether/ethyl acetate/methanol 3:1:0.5) 0.115 g (69%) of **3e** was obtained; viscous oil; IR (KBr): *υ*_max_ 3354, 3229, 3061, 3027, 2928, 2862, 1665, 1607, 1590, 1526, 1504, 1469, 1285, 1198, 838, 670 cm^–1^. MS/MS *m/z* 334.2 (M+H)^+^. Anal. (C_20_H_19_N_3_O_2_) C, H, N.

#### 3.1.4.General Procedure for the Synthesis *of* 4-[4-[[4-Chloro-3-(trifluoromethyl)phenyl]carbamoyl-amino]phenoxy]-pyridine-2-carboxamides4 **a–e**

A solution of 4-(4-aminophenoxy)-picolinamide **3a**–**e** (0.1 mmol) and 4-chloro-3-(fluoromethyl)-phenyl isocyanate (0.12 mmol) in anhydrous dichloromethane (3 mL) was stirred for 16 h at room temperature under nitrogen atmosphere. Solvent was evaporated under reduced pressure and the residue was dissolved in ethyl acetate and extracted three times with brine. The organic phase was dried over anhydrous Na_2_SO_4_, filtered and evaporated under reduced pressure. The crude products **4a**–**e** were purified by column chromatography, preparative chromatography or by recrystallization.

*4-[4-[[4-Chloro-3-(trifluoromethyl)phenyl]carbamoylamino]phenoxy]-N-cyclopentyl-pyridine-2-carboxamide* (**4a**). From the reaction of compound **3a** (0.030 g, 0.1 mmol) and purification by column chromatography (mobile phase petroleum ether/ethyl acetate/methanol 3:1:0.5) 0.051 g (98%) of **4a** was obtained; viscous oil; IR (KBr): *υ*_max_ 3353, 2962, 2872, 1718, 1654, 1595, 1546, 1506, 1420, 1306, 1199, 1176, 1140, 838, 663 cm^–1^. MS/MS *m/z* 519.1 (M+H)^+^. Anal. (C_25_H_22_ClF_3_N_4_O_3_) C, H, N.

*4-[4-[[4-Chloro-3-(trifluoromethyl)phenyl]carbamoylamino]phenoxy]-N-cyclohexyl-pyridine-2-carboxamide* (**4b**). From the reaction of compound **3b** (0.031 g, 0.1 mmol) and purification by column chromatography (mobile phase petroleum ether/ethyl acetate/methanol 3:1:0.5) and preparative chromatography (mobile phase dichloromethane/methanol 9:1) 0.023 g (43%) of **4b** was obtained; mp 110-114 °C; IR (KBr): *υ*_max_ 3348, 3072, 2932, 2855, 1716, 1650, 1596, 1548, 1505, 1484, 1419, 1293, 1197, 1175, 1137, 840, 663 cm^–1^. MS/MS *m/z* 533.2 (M+H)^+^. Anal. (C_26_H_24_ClF_3_N_4_O_3_) C, H, N.

*4-[4-[[4-Chloro-3-(trifluoromethyl)phenyl]carbamoylamino]phenoxy]-N-cyclohexylmethyl-pyridine-2-carboxamide* (**4c**). From the reaction of compound **3c** (0.033 g, 0.1 mmol) and purification by column chromatography (mobile phase petroleum ether/ethyl acetate 1:1) and recrystallization from methanol/water 0.030 g (55%) of **4c** was obtained. mp 156-160 °C; IR (KBr): *υ*_max_ 3353, 2962, 2872, 1718, 1654, 1595, 1546, 1506, 1420, 1306, 1199, 1176, 1140, 838, 663 cm^–1^. MS/MS *m/z* 547.2 (M+H)^+^. Anal. (C_27_H_26_ClF_3_N_4_O_3_) C, H, N.

*4-[4-[[4-Chloro-3-(trifluoromethyl)phenyl]carbamoylamino]phenoxy]-N-benzyl-pyridine-2-carbox-amide* (**4d**). From the reaction of compound **3d** (0.032 g, 0.1 mmol) and recrystallization from methanol/water 0.020 g (37%) of **4d** was obtained. mp 160-163 °C; IR (KBr): *υ*_max_ 3351, 3132, 2926, 2854, 1718, 1654, 1595, 1543, 1506, 1420, 1303, 1199, 1176, 1140, 837, 668 cm^–1^. MS/MS *m/z* 541.9 (M+H)^+^. Anal. (C_27_H_20_ClF_3_N_4_O_3_) C, H, N.

*4-[4-[[4-Chloro-3-(trifluoromethyl)phenyl]carbamoylamino]phenoxy]-N-phenylethyl-pyridine-2-carboxamide* (**4e**). From the reaction of compound **3e** (0.033 g, 0.1 mmol) and purification by column chromatography (mobile phase petroleum ether/ethyl acetate/methanol 3:1:0.15) 0.041 g (74%) of **4e** was obtained. viscous oil; IR (KBr): *υ*_max_ 3345, 3066, 2928, 1716, 1654, 1596, 1545, 1505, 1483, 1419, 1299, 1198, 1175, 1134, 840, 699 cm^–1^. Anal. (C_28_H_22_ClF_3_N_4_O_3_) C, H, N.

### 3.2. Biological Evaluation

#### 3.2.1. Cytostatic Activity

HCT 116, SW 620, MCF-7, and H 460 cells were cultured as monolayers and maintained in Dulbecco’s modified Eagle medium (DMEM), supplemented with 10% fetal bovine serum (FBS), 2 mmol·L^–1^ L-glutamine, 100 U·mL^–1^ penicillin and 100 μg·mL^–1^ streptomycin in a humidified atmosphere with 5% CO_2_ at 37 °C. The growth inhibition activity was assessed as described previously, according to the slightly modified procedure of the National Cancer Institute, Developmental Therapeutics Program [14,15]. Briefly, the cells were seeded onto standard 96-well microtiter plates on day 0. The cell concentrations were adjusted according to the cell population doubling time (PDT): 3000 cells/well for HCT116 and SW 620 cell lines (PDT = 20–24 h) and 4,000 cells/well for MCF-7 cell lines (PDT = 33 h). Tested agents were then added in five consecutive 10-fold dilutions (10^–8^ to 10^–4^ mol·L^–1^) and incubated for further 72 h. Working dilutions were freshly prepared on the day of testing. The solvent (DMSO) was also tested for eventual inhibitory activity by adjusting its concentration to be the same as in working concentrations (maximal concentration of DMSO was 0.25%). After 72 h of incubation, the cell growth rate was evaluated by performing the MTT assay (Promega) which detects dehydrogenase activity in viable cells [16]. The absorbency (OD, optical density) was measured on a microplate reader at 570 nm. Each test point was performed in quadruplicate in at least three individual experiments. The results are expressed as IC_50_, which is the concentration necessary for 50% of inhibition. The IC_50_ values for each compound are calculated from dose-response curves using linear regression analysis, using FORCAST function in Excel, by fitting the test concentrations that give PG (percentage of growth) values above and below the reference value (*i.e.*, 50%). Each result is a mean value from three separate experiments.

The cytostatic activity of the tested compounds against murine leukemia L1210, human cervix carcinoma HeLa, human embrionic lung (HEL) and T-lymphocyte CEM cells was determined with cells grown in suspension (L1210, CEM) or monolayers (HeLa, HEL) in 200 µL-wells of 96-well microtiter plates (initial cell number: 5–7.5 × 10^4^ cells/well). After 48 (L1210) or 72 (CEM, HeLa, HEL) h, the tumour cell number was determined by the use of a Coulter counter.

#### 3.2.2. Antimetabolic Activity and Drug Influx/Efflux Assay in CaCo-2 Cell Cultures

The procedures have been modified from previously described methods [12,13]. Human CEM cells were seeded in 96-well microtiter plates at 10 × 10^4^ cells/200 µL-well in the presence of serial dilutions of the tested compounds. Then, 0.25 µCi of [CH_3_-^3^H]dThd (radiospecificity: 52 Ci/mmol) or 1 µCi of [5-^3^H]Urd (radiospecificity: 26 Ci/mmol) or [4,5-^3^H]Leu (radiospecificity: 160 Ci/mmol) were added and the drug-exposed tumour cell cultures further incubated at 37 °C for ~20 h. Then the content of each well was transfered to glass fiber discs (Wattman, GE Healthcare, Maidstone, UK), mounted on a manifold (Millipore, Billerica, MA, USA) and subsequently washed with PBS (to remove the radiolabel material that has not been incorporated in the cellular macromolecules), 10% and 5% trichoroacetic acid (TCA) (to precipitate DNA, RNA and proteins) and ethanol. Then, the radioactivity (TCA precipitate) associated with each disc was determined in a liquid scintillation counter (Packard, Palo Alto, CA, USA).

Human colon carcinoma CaCo-2 cells were seeded in the inner wells of a double-chamber-well-tray at 150,000 cells (0.5 mL) per 1 mL-well. The outer wells also contain 0.5 mL medium (without cells). After the cells were grown to confluency (~2 days) at 37 °C in a humidified CO_2_-controlled atmosphere, 100 µmol·L^–1^ tested compound (final concentration) was added to the inner well, and the medium from the outer wells was replaced by 800 µL PBS (containing 10% foetal bovine serum). At different time points (*i.e.*, 0, 2, 4, 6, 8 and 24 h), 50 µL was removed from the outer wells and mixed with 100 µL cold methanol. The mixture was left on ice for 10 min, centrifuged to remove the precipitate, and the supernatant was analysed by HPLC (reversed phase) to determine the drug concentration that appeared in the outer wells (reflecting basolateral drug efflux) in function of time.

## 4. Conclusions

The synthetic pathway leading to five new sorafenib amide derivatives is described. The final compounds, as well as intermediates, are fully chemically characterized. Their cytostatic activity was tested on a panel of seven tumour cell lines, on which they exerted pronounced, but nonselective antiproliferative activity at the lower micromolar range, comparable to the activity of sorafenib itself. Detailed pharmacological and pharmacokinetic evaluation still remain to be done to compare the sorafenib derivatives described herein with the parent compound.
